# Underneath the Gut–Brain Axis in IBD—Evidence of the Non-Obvious

**DOI:** 10.3390/ijms252212125

**Published:** 2024-11-12

**Authors:** Lidiya V. Boldyreva, Anna A. Evtushenko, Maria N. Lvova, Ksenia N. Morozova, Elena V. Kiseleva

**Affiliations:** 1 Scientific-Research Institute of Neurosciences and Medicine, 630117 Novosibirsk, Russia; evtushenkoaa@neuronm.ru; 2 Institute of Cytology and Genetics, The Siberian Branch of the Russian Academy of Sciences, 630090 Novosibirsk, Russia; lvovamaria@bionet.nsc.ru (M.N.L.); morozko@bionet.nsc.ru (K.N.M.); elka@bionet.nsc.ru (E.V.K.)

**Keywords:** gut–brain axis, IBD, inflammation, metabolome, lipidome, mitochondrial function, cationic transport, cytoskeleton

## Abstract

The gut–brain axis (GBA) plays a pivotal role in human health and wellness by orchestrating complex bidirectional regulation and influencing numerous critical processes within the body. Over the past decade, research has increasingly focused on the GBA in the context of inflammatory bowel disease (IBD). Beyond its well-documented effects on the GBA–enteric nervous system and vagus nerve dysregulation, and gut microbiota misbalance—IBD also leads to impairments in the metabolic and cellular functions: metabolic dysregulation, mitochondrial dysfunction, cationic transport, and cytoskeleton dysregulation. These systemic effects are currently underexplored in relation to the GBA; however, they are crucial for the nervous system cells’ functioning. This review summarizes the studies on the particular mechanisms of metabolic dysregulation, mitochondrial dysfunction, cationic transport, and cytoskeleton impairments in IBD. Understanding the involvement of these processes in the GBA may help find new therapeutic targets and develop systemic approaches to improve the quality of life in IBD patients.

## 1. Introduction: The GBA Imbalance in IBD

The gut–brain axis (GBA) is a complex and bidirectional communication system driven by neural, hormonal, metabolic, immunological, and microbial signals, playing a crucial role in linking the state of the gastrointestinal tract with neural functions. The GBA can influence intestinal motility and secretion, cause visceral hypersensitivity, and induce cell modifications in the enteroendocrine and immune systems [1,2]. Numerous neurological disorders have been shown to be associated with changes in gut health [3,4]. Systematic data have shown that both brain–gut and gut–brain dysfunctions occur, with the former being particularly dominant in irritable bowel syndrome [5]. In patients with inflammatory bowel diseases (IBDs), the quality of life is significantly reduced, and eating behaviors and nutritional patterns often undergo inevitable changes [6,7]. IBD is characterized by chronic inflammation of the gastrointestinal tract, encompassing Crohn’s disease and ulcerative colitis, which are differentiated by the location of the lesions. Crohn’s disease is marked by inflammation and lesions along the entire length of the gastrointestinal tract, predominantly in the terminal ileum and proximal colon [8], whereas ulcerative colitis mainly affects the colon and rectum [9]. Regardless of injury location, both diseases share common symptoms: persistent diarrhea, abdominal pain, blood in stools, weight loss, and chronic fatigue [8,10]. Various factors contribute to the development of IBD: genetic predispositions (with several hundred associated genes identified [11,12,13,14,15,16,17,18]), immune response dysregulation [19,20,21,22], impaired intestinal microbiota (due to antibacterial and other drug administrations or specific dietary patterns) [23,24,25], and changes in environmental factors (such as nutrition, stress, toxins, etc.) [26]. IBD is a serious global issue, as the number of diagnosed patients continues to rise [27,28,29]. Increasing evidence suggests a connection between IBD and neuroinflammatory as well as neurodegenerative diseases [30,31]. According to the meta-analysis results published in 2024, IBD is moderately associated with an increased risk of stroke, dementia of various etiologies, and Parkinson’s disease [32]. In addition, the risk for the development of depression and anxiety in patients and animal models with IBD has been found higher compared to the general population, and stress is a significant trigger that causes exacerbation of the disease [33,34]. Moreover, IBD-related negative effects including intestinal inflammation, dysbiosis, poor nutrition, physical and psychical discomfort, chronic fatigue, and stress are frequently self-reinforcing due to the GBA imbalance resulting from IBD [34,35,36].

### 1.1. The Current Gut–Brain Axis Paradigm

The GBA commonly accepted key components include the vagus nerve, the enteric nervous system (ENS), and the gut microbiota. The vagus nerve serves as the primary neural pathway connecting the gut and the brain, comprising sensory and motor fibers that facilitate bidirectional communication [37]. Sensory fibers transmit impulses from the gut to the brain, relaying signals related to satiety, nutrient availability, and gastrointestinal discomfort, while motor fibers convey instructions from the brain to modulate gut functions such as gastric secretion and motility [38]. This bidirectional communication ensures continuous feedback and regulation between the gut and the brain. The ENS is an extensive network of intrinsic neurons throughout the gastrointestinal tract, functioning independently of the central nervous system (CNS) and regulating essential gastrointestinal processes, including peristalsis, nutrient absorption, and gut motility [39]. Although numerous studies highlight the evident disruption of vagus nerve and ENS functions in IBD [40,41,42], the underlying molecular and cellular processes, as well as the involved signaling triggers, remain underexplored. In the following sections, we describe the relevant data on the processes significantly disrupted in IBD that have yet to be investigated in the context of GBA dysregulation. These underexplored areas may hold the key to understanding the intricate mechanisms linking gut and brain health in IBD.

The gut microbiota, a diverse community of microorganisms within the gastrointestinal tract, plays beneficial roles in metabolism, vitamin and essential metabolite synthesis, and pathogen inhibition [43,44,45]. As the result of inflammation in IBD, as a consequence of specific IBD treatments, gut microbiota undergoes dramatic changes, observed in both patients and animal models [46,47]. An imbalance in the gut microbiota reflected in the disproportion between pathogenic and symbiotic bacteria may occur in addition to the loss of bacterial diversity calling dysbiosis. Dysbiosis, through effects on certain cytokines like tumor necrosis factor, interleukin 6, and interleukin beta, can induce an inflammatory process associated with an increase in the permeability of the intestinal barrier due to the depletion of apical junction complexes’ (AJCs) proteins [48,49,50]. Animal and human studies have shown that dysbiosis can affect the onset and progression of IBD, as well as autism, diabetes, Alzheimer’s, and Parkinson’s diseases [51,52,53,54,55,56,57]. Animal studies have shown that microbiota can significantly impact brain function and behavior, with “germ-free” mice displaying deviations in brain volume, myelination, as well as CNS deficits, such as anxiety-like behaviors, memory and sociability impairments, and depressive symptoms [58,59,60]. Both animal and human studies demonstrate that individuals with depression often present a microbial imbalance characterized by reduced microbial abundance and species diversity. It has been demonstrated that the transfer of microbiota from patients with depression to rats induces depression-like behavior along with alterations in tryptophan metabolism in the recipient animals [61]. In another experiment involving the transfer of microbiota from patients to “germ-free” mice, not only were neurobehavioral changes observed, but there was also evidence of shifts in metabolic regulation [62]. Changes in the microbiota composition have been observed in patients with autism, and the ability of fecal supernatant from autism spectrum disorder patients to induce gastrointestinal epithelium and ENS malfunctions in mice has also been demonstrated [63,64]. Therefore, symbiotic and pathogenic microbiota can also be involved in the GBA by producing biologically active metabolites: short-chain fatty acids and their precursors, particular neurotransmitters, and a variety of signaling molecules that can influence neural activity, immune response, as well as other physiological processes within the host organism [65,66]. In this review, we compile evidence regarding the currently underrated processes through which microbiota may contribute to the GBA imbalance in the course of IBD pathogenesis.

### 1.2. Diet Effects and Food Behavior in IBD

Studies exploring the involvement of diet in IBD patients have uncovered a significant impact of dietary preferences and food behavior both on neural functions and IBD progression. IBD patients with high food involvement tend to seek pleasurable food; however, individuals with high health engagement levels have a better emotional state and demonstrate lower hospitalization rates and relapses [7,67]. At the same time, avoidant eating behaviors common in IBD patients pose a high risk of avoidant/restrictive food intake disorder (ARFID). This, along with impaired absorption of nutrients in IBD, can result in supplemental deficits, digestive malfunction, and metabolic dysregulation [68]. Murine studies have found dietary effects on intestinal microbial diversity associated with changes in cognitive ability and behavior [69,70,71] and similar observations have been made in human studies [72,73]. Specific dietary schemes are now widely discussed as an approach to ameliorate mental health, with growing evidence from preclinical and clinical studies of the dietary impact on neurologic and psychiatric disorders, including neuroinflammation, cognitive decline, depression, autism spectrum disorder, epilepsy, Alzheimer’s, and Parkinson’s diseases [74,75].

Thus, the interrelation between gut microbiota, diet, and metabolism represents a finely balanced equilibrium that not only plays an essential role in the overall health but also in brain physiology, influencing behavior and mental health. In IBD, this equilibrium is inevitably imbalanced, along with chronic and recurrent inflammatory processes. Together, these factors lead to malfunctions of fundamental cellular mechanisms such as metabolic homeostasis, ion transport, and cytoskeletal and mitochondrial functions. In turn, these profound changes make IBD difficult to treat and cause associated disease complications, substantially involving GBA dysregulation [76,77]. The GBA in IBD has been the focus of recent research, with accumulating data on the impact of main IBD-related processes on the GBA, including inflammation, microbiome, and vagus nerve and ENS functions. At the same time, cellular and molecular processes essential for nervous system function that are impaired in IBD remain largely unexplored.

**Figure 1:**
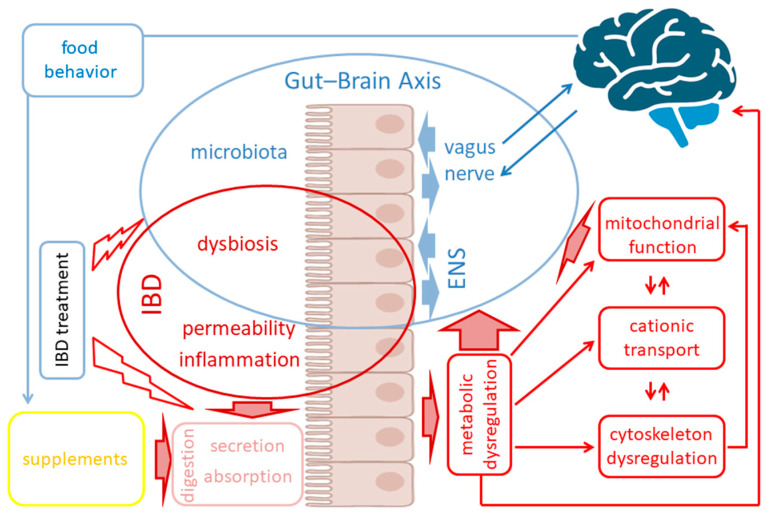
Schematic overview of metabolic dysregulation, mitochondrial function, cationic transport, and cytoskeletal dysregulation roles within GBA imbalance in IBD pathogenesis. Blue arrows indicate normal interactions; red arrows and broken arrows indicate negative effects of IBD-related processes and IBD treatment, accordingly.

The purpose of this review is to summarize research data on metabolic homeostasis, mitochondrial function, ion transport, and cytoskeleton function involvement in IBD-related pathological processes concerning GBA imbalance (Figure 1) and consider in detail the possible pathways and cellular and molecular mechanisms that link these effects to each other, leading to the self-exacerbation of the negative effects of IBD. This review intends to put a research focus on these currently underexplored areas in regard to GBA. We believe that elucidating the role of these mechanisms in the GBA could open prospects for new systemic therapeutic approaches in IBD.

## 2. Metabolic Dysregulation

**Figure 2:**
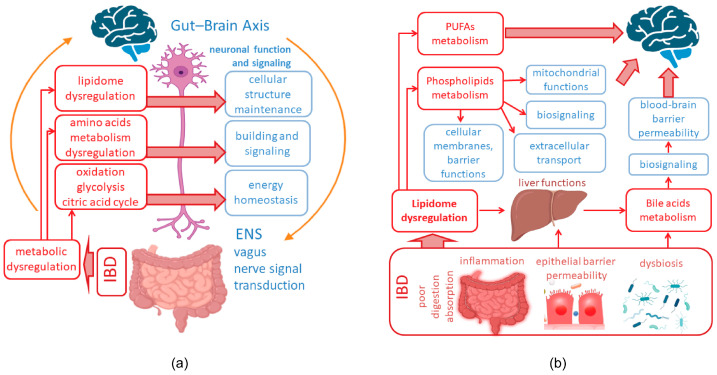
Schematic overview of mechanisms of metabolic dysregulation impact on GBA functions in IBD. (**a**) Schematic presentation of IBD-induced metabolic dysregulation effects on neural cells’ functions and signaling within GBA. Red boxes indicate metabolic processes found impaired in IBD; blue boxes indicate neural cellular functions subject to metabolic dysregulation effects; red arrows indicate negative effects of IBD-related processes; (**b**) Schematic presentation of IBD-related lipidome dysregulation effects on cellular and organelle membranes and functions; biosignaling processes; and extracellular transport and blood–brain barrier permeability. Red boxes indicate classes of compounds whose metabolism is impaired in IBD; blue boxes indicate cellular functions subject to the corresponding lipidome dysregulation effects; red arrows indicate negative effects of lipidome dysregulation.

In IBD patients, significant alterations in metabolic processes and pathways are commonly observed [78,79,80]. Metabolic profiling is currently being explored as a method for early identification of IBD [79,80,81]. Specific interrelationships have been established between changes in metabolite levels in IBD and the induction and development of immune responses, oxidative process dysregulation, mitochondrial malfunction [82,83,84,85], and remarkable lipidome deregulation [86,87]. In animal models of colitis, mass spectrometry assays have also revealed substantially altered lipid ratios and levels of enzymes that regulate major metabolic signaling cascades, including oxidative stress, β-oxidation, glycolysis, and the citric acid cycle [88,89,90,91,92] (Figure 2a). The lipidome homeostasis is fundamental to neural cell function and the nervous system makes great use of all classes of lipids (fatty acids, triglycerides, phospholipids, sterol lipids, and sphingolipids) and also contains the greatest proportion of lipids in the human body [93]. Lipids also play a key role in maintaining total metabolic homeostasis through cellular processes mediated by leukotriene, prostanoid, and endocannabinoid signaling; they also attenuate inflammation, regulate metabolic diseases, and control physiological processes [94,95,96,97,98]. The involvement of fatty acids and their derivatives in intracellular signaling is well documented [99,100,101]. Since phospholipids are main structural components of cellular and organelle membranes, dysregulation of the lipidome may contribute to mitochondrial dysfunction via membrane structure impairments [102,103,104]. Transmembrane proteins’ conformation and functionality, including receptors and ion channels’, are also highly dependent on membrane lipid composition [104,105]. Therefore, regarding metabolic dysregulation in IBD, the lipidome imbalance may play a key role in GBA dysregulation (Figure 2b). Despite the critical role of lipid metabolism and energy homeostasis in neural functions, the involvement of these processes in GBA dysregulation in IBD remains poorly understood.

### 2.1. PUFAs’ Role and Therapeutic Potential in GBA Balance Restoration in IBD

Among major classes of lipids in CNS, polyunsaturated fatty acids (PUFAs) have the most well-defined regulatory role. n-3 PUFAs have been shown to be a limiting factor for proper neurodevelopment during the perinatal period [106]. PUFAs are implicated in neuronal signaling, neurogenesis control, vesicular processes, and central glucose homeostasis and are known for their ability to affect the mood and cognition [101,107]. Neural tissue is enriched with two major PUFAs, arachidonic acid and docosahexaenoic acid, which are crucial for brain development and neural functions [108,109,110]. PUFAs’ primary targets are fatty acid-activated receptors, the most studied of which are the peroxisome proliferator-activated receptors (PPARs). PPARβ and PPARδ in the brain have been shown to regulate inflammatory responses and fatty acid metabolism [111]. PUFAs’ involvement is also known in multiple distinct signaling pathways in neural cells. Anandamide and 2-arachidonoylglycerol, the arachidonic acid derivatives, are the major forms of endocannabinoids in the brain and are known to bind to cannabinoid receptor types 1 and 2 both in neuronal and glial cells to suppress neurotransmitter release [112,113]. This has been shown to mediate short-term synaptic plasticity and long-term depression in excitatory and inhibitory synapses [114]. A relationship between n-3 PUFAs and the gut microbiota has been shown: n-3 PUFAs intake alters the abundance and species composition of the microbiome. An imbalanced consumption of n-3/n-6 PUFAs in the diet can induce dysbiosis, in particular a significant increase in the *Firmicutes*/*Bacteroidetes* ratio, which was shown to be associated with metabolic imbalance [115]. An n-3 PUFA deficit has been shown to impair the microbiota composition in metabolic disorders. Conversely, the gut microbiota can also significantly affect the metabolism and absorption of PUFAs [116].

The beneficial effects of PUFAs on the GBA balance have been demonstrated in both murine models and clinical trials, leading to their consideration as a supportive supplement in the complex therapy of neuropsychiatric pathologies [117,118]. In IBD, the anti-inflammatory effects of PUFAs have been observed in murine colitis models [119,120,121], along with a preventive effect on liver inflammation and oxidative stress [120,122]. However, clinical studies on the effects of PUFAs intake in IBD have produced contradictory results [123,124]. PUFAs likely exert an indirect regulatory effect on IBD-related processes [125] and are possibly involved in the complex regulation of lipid homeostasis. Thus, PUFAs have been shown to downregulate Sterol Regulatory Element Binding Protein 1 (SREBP1) activity, which is a crucial actor in mitochondrial lipogenesis [126]. Studying specific PUFA supplements that may benefit GBA in IBD and understanding the involved regulatory pathways appear to be a promising direction for the research and therapeutic implication.

### 2.2. Phospholipid Involvement in GBA Misbalance in IBD

Lipidome dysregulation can lead to cellular membrane damage, as well as significant changes in crucial cell signaling, excitotoxicity due to excitatory amino acid production rise, and increased apoptosis [94]. A growing body of evidence highlights the importance of phospholipid (PL) metabolism dysregulation in the pathological changes associated with neuronal functions [127,128]. Dysregulation of PL metabolism is implicated in Alzheimer’s and Parkinson’s diseases, as well as other neural pathologies [102,129]. At the same time, PL play a significant role in the pathological processes related to IBD [130]. Inflammation-induced cell membrane destruction, a common trait of IBD, can affect both enterocytes and ENS cells during both acute and chronic phases of IBD. Consequently, receptor-mediated pathways involving PL cleavage into secondary messengers may substantially contribute to the GBA imbalance in IBD.

The catabolism of the major membrane phospholipid, phosphatidylcholine (PC), is associated with crucial regulatory pathways in both enteric epithelial and neural cells [130,131,132]. PC was found to have an effect in the IBD treatment by the gut barrier function modulation, polarization of macrophages regulation, and by reducing the inflammation and is also known to have a potential in remodeling gut microbiota [133,134,135]. Concurring mechanisms are known for PC hydrolysis, including protein kinase C (PKC)-dependent and independent pathways. The activation of M-cholinergic receptors due to an increased need for choline, the precursor of acetylcholine, induces the stimulation of PC-sensitive phospholipase D [136]. Cholinergic neuron hyperactivation reduces the concentration of extracellular choline, potentially limiting its entry into neighboring neurons and interfering with the synthesis of membrane PC [132]. Additionally, phospholipase D activation is mediated by adrenergic stimulation [137], glutamate, and metabotropic receptors’ stimulation [138]. The PKC impact on phospholipase D catalytic activity is dual: stimulation via an ATP-independent pathway or inhibition under phosphorylation [139,140]. PC can also be utilized via the activation of phospholipase A2 that is mediated by the glutamate effect on NMDA receptors [141,142]. Notably, NMDA receptor stimulation, which mediates the cleavage of choline from PC, prevents its reutilization into PC by inhibiting choline phosphotransferase activity. This pathway of glutamatergic activation for acetylcholine synthesis can precede neuronal death [138]. Therefore, PC is a crucial component and biochemical regulator of neuronal functions, and its impaired levels and catabolism in IBD may act as a trigger in GBA imbalance.

Phosphatidylinositol (PI) and its metabolites also play a crucial role in neuronal functions, orchestrating the membrane transport of mediators, including processes like endocytosis, exocytosis, and vesicle binding [136,143]. PI metabolism and the formation of its derivatives have been shown to be impaired in IBD both in patients and in animal models [144,145,146]. Phosphorylated forms of PI are crucial regulators in neurotransmission through the mediators’ release from presynaptic membranes modulation acting on postsynaptic receptors [147]. At least 15 cytoskeletal actin-binding proteins have been reported to interact with or being regulated by phosphoinositides, whose synthesis is regulated by extracellular signals [148]. Hyperinduction of phosphoinositides hydrolysis leads to calcium transport impairments that can result in neuronal damage and death [149]. The decrease in neuronal level PI metabolite, PI-(4,5)-diphosphate, enhances the formation of β-amyloid from APP, and this process is Ca^2+^-dependent [150]. PI-(3,4,5)-trisphosphate has been found to be reduced in the brain of Alzheimer’s disease patients and in mouse models, in association with endosomal system impairments [151]. Conversely, β-amyloid is known to affect transmembrane signaling, affecting the metabolism of PI and PI-(4,5)-diphosphate. PI and PI-(4)-monophosphate are thought to perform a neuroprotective role, since their addition to the culture of hippocampal neurons eliminated the toxic effect of β-amyloid [152]. Low concentrations of β-amyloid peptide were shown to stimulate, but high concentrations to inhibit, phospholipase C activity [153]. Decreased phospholipase C expression in the brain is associated with the terminal stage of dendrite degradation [154]. Catabolism of PI is under the control of phospholipase A2, which exhibits activity both under increased Ca^2+^ concentration (Ca^2+^-dependent phospholipase A2) or in Ca^2+^ absence, the latter being limited by PL metabolites (Ca^2+^-independent phospholipase A2) [155]. The activation of phospholipase A2 is responsible for the biosynthesis of secondary PL messengers: arachidonic acid, eicosanoids, PAF, and diglycerides [156]. Phospholipase A2 is also involved in processes associated with increased dendritic growth and neuronal differentiation during the nervous system development, as well as involved in cognitive functions [157,158].

Dietary modulation and therapeutic approaches towards normalizing PL metabolism, as far as the PL membrane composition maintaining the organelle health, may emerge as a novel approach in the systemic therapy of IBD and its related effects on the GBA.

### 2.3. Bile Acids’ Shift in IBD and Their Regulatory Potential in GBA

Bile acids (BAs) are among key metabolic regulators of microbial origin in the intestine; primary bile acids, cholic acid (CA) and chenodeoxycholic acid (CDCA), are synthesized in liver hepatocytes from cholesterol. The intestinal microflora is responsible for deconjugation, desulfation, conjugation with amino acids, and the formation of secondary BAs in the intestinal lumen [159,160]. Bacteria belonging to the phylum *Firmicutes*, such as *Clostridium cluster XIVa*, are responsible for the production of secondary BAs [161,162]. BAs perform a range of regulatory functions, acting as signaling molecules in cholesterol, lipid, steroid, and glucose metabolic cycles [163]. Changes in the composition of the microbiota and its enzymatic activity, characteristic of IBD, lead to alterations in the luminal pool of BAs and the range of functions they perform; this contributes to the maintenance of chronic inflammation and the formation of metabolic disorders [164,165,166,167,168] (Figure 2b). BA metabolism and signaling are substantially altered in IBD, with bacteria producing significant amounts of unusual BAs, whose roles remain to be fully elucidated [165,169]. In IBD patients, particularly those with Crohn’s disease, the impairments in the deconjugation, transformation, and desulfation activities of the gut microbiota are observed. This disruption leads to increased levels of sphingolipids and the primary bile acids CA and CDCA in the stool, alongside a depletion of secondary bile acids (lithocholic acid (LCA) and deoxycholic acid (DCA)) [170]. Significant reductions in secondary BAs (LCA and DCA) have been observed in the stool and serum samples of Crohn’s disease patients with psychological disorders. These patients’ stool samples showed an enrichment of primary BAs (CA and CDCA), while their serum exhibited elevated concentrations of dehydrocholic acid (DHCA) compared to healthy volunteers [171]. Additionally, levels of taurodeoxycholic acid (TDCA), taurolithocholic acid (TLCA), and tauro-β-muricholic acid (TβMCA) in feces positively correlated with anxiety scores [171,172]. High levels of sulfated fatty acids have also been identified in the feces of IBD patients, a characteristic trait of the disease [165]. Furthermore, Crohn’s disease patients exhibit elevated levels of BAs conjugated with phenylalanine, tyrosine, or leucine [160]. Comprehensive omics analyses of fecal samples from patients with various types of IBD have shown shifts in bacterial taxa with marked sensitivity to BA-rich environments, including *Faecalibacterium prausnitzii, Gammaproteobacteria u Blautia* spp. [173,174]. IBD patients with significant depression or anxiety displayed markedly reduced fecal microbiota diversity and lower counts of *Faecalibacterium prausnitzii* compared to patients without psychological disorders [171]. Studies in rats have demonstrated that these bacteria possess therapeutic effects in alleviating depressive and anxious states, highlighting their potential role in managing psychological symptoms in IBD patients [175]. Recent clinical findings have confirmed the involvement of gut microbiota dysbiosis and altered BA metabolism in the psychological disorders associated with Crohn’s disease [171].

BAs have been shown to penetrate the blood–brain barrier [176], and numerous studies have identified both primary and secondary BAs in the brains of humans and rats [177,178,179,180]. The unconjugated bile acids CA, CDCA, DCA, and ursodeoxycholic acid (UDCA) can pass through the phospholipid bilayer of the blood–brain barrier due to their hydrophobic properties [173,177], and their concentrations in the brain correlate with their concentrations in serum [141]. Conjugated BAs, due to their greater hydrophilicity and size, can pass through the blood–brain barrier via active transport. Expression of mRNAs of various bile acids transporters was found in the vascular plexus of rat brain ventricles [181]. It was shown that the increase in BA concentration in the blood serum in rats after bile duct ligation or intravenous injection of DCA and CDCA caused structural damage, increased blood–brain barrier permeability, and increased concentration of these BAs in the brain [182,183]. In vitro studies have demonstrated that BAs can disrupt tight junctions in monolayers of rat brain microvascular endothelial cells by causing phosphorylation of occludins and increasing permeability via a Rac1-dependent pathway [182]. In a mouse model of non-alcoholic steatohepatitis (NASH) combined with moderate chronic colitis, alterations in the composition and levels of BAs were observed. These changes were accompanied by an increase in the protein S100β, a marker of brain permeability and glial cell activation, in the blood serum, along with a significant reduction in the tight junction proteins ZO-1 and occludins [184].

The most extensively studied bile acid receptors are the nuclear receptor farnesoid X receptor (FXR) and the membrane-bound receptor G protein-coupled bile acid receptor 1 (GPBAR1), also known as Takeda G protein-coupled receptor 5 (TGR5) [185,186]. These receptors are highly conserved in humans and mice [187]. FXR is the primary regulator of BA synthesis and enterohepatic circulation. The signaling pathways and molecular mechanisms through which FXR and TGR5 interact with BAs in the enterohepatic organs are comprehensively reviewed in [188]. These and other BA receptors have also been identified in the brains of humans and rodents [189]. Studies on FXR knockout mice have demonstrated that FXR plays a role in the homeostasis of glutamatergic, GABAergic, serotonergic, and noradrenergic neurotransmitter systems in the hippocampus and cerebellum, influencing locomotor and cognitive functions as well as behavior [190]. In mouse models of hepatic encephalopathy, the activation of FXR receptors in brain neurons by bile acids is associated with neurological decline [191]. Increased expression of FXR in the hippocampus of naïve rats induces depressive-like behavior and reduces the expression of brain-derived neurotrophic factor (BDNF) [192]. Conversely, the knockdown of hippocampal FXR completely prevents the negative effects of chronic unpredictable mild stress on rat behavior [192]. In cell culture studies, conjugates with the amino acids phenylalanine, tyrosine, or leucine have been identified as potent agonists of the human FXR. These conjugates are comparable in efficacy to CDCA, the most powerful natural FXR agonist. It was shown that the level of BAs conjugated with the three listed amino acids was significantly higher in the dysbiotic state of patients with Crohn’s disease but not in patients with ulcerative colitis [160]. When mice were fed these compounds, there was a notable reduction in the expression of downstream FXR target genes responsible for bile acid synthesis in the liver. [160]. In the brain, the bile acid receptor TGR-5 is present on neurons, astrocytes, and microglial cells. Studies on a mouse model of hepatic encephalopathy, as well as in vitro studies on primary neurons and the mouse microglia cell line (EOC-20), have shown that the central activation via intracerebroventricular infusion of a TGR-5 agonist leads to reduced neuronal expression of chemokine ligand 2 (CCL-2), decreased production of pro-inflammatory cytokines, and reduced microglial proliferation [193]. Both in vitro and in vivo studies in mouse models of acute neuroinflammation have demonstrated that the binding of tauroursodeoxycholic acid (TUDCA) to TGR-5 exerts anti-inflammatory effects, influences microglial phenotype, reduces NFκB activation, and induces the TGFβ pathway [194]. Another bile acid receptor, sphingosine-1-phosphate receptor (S1PR2), is expressed by endothelial cells and neurons. In mice, S1pr2−/− knockout showed weaker disruption of the blood–brain barrier and reduced neutrophil infiltration during systemic inflammation induced by lipopolysaccharide compared to S1pr2+/− littermates, indicating S1PR2’s role as a mediator of cerebrovascular inflammation [195]. In an azoxymethane-induced mouse model of hepatic encephalopathy, high levels of the conjugated primary bile acid taurocholic acid in the blood led to the activation of S1PR2 in brain neurons (cortex and hippocampus), resulting in increased expression of mRNA and secretion of CCL2. This activation subsequently stimulated microglia, leading to increased expression of pro-inflammatory cytokines and sustained neuroinflammation [196]. The specific ways of brain-localized bile acid receptors’ contribution to neurological changes remain largely unclear. However, many of these receptors are considered potential therapeutic targets for treating neurodegenerative diseases and psychiatric disorders [194,195,197,198].

Recently, the first clinical data confirming the involvement of gut dysbiosis and BA metabolism alteration in psychological disorders in Crohn’s disease were obtained [171]. As our understanding of BAs’ roles in neural functions grows, they are becoming perspective actors in the gut–brain axis in the context of IBD and gut microbiota misbalance. 

Metabolic dysregulation is fundamentally involved in IBD and should be considered in relation to the GBA dysregulation. Currently, data are fragmentary, and specific pathways are poorly understood, necessitating a systematic approach to identify the metabolic pathways disrupted in IBD and their involvement in the GBA, as far as targeted strategies for their normalization are concerned. A promising avenue in diagnostics is a personalized metabolomics analysis approach. Potential methods for metabolic correction may include (i) the therapeutic and dietary compensation of supplementary deficiencies, addressing specific metabolic deficits defined, and (ii) the development of pharmaceuticals to modulate metabolic pathways and restore metabolic homeostasis.

## 3. Mitochondrial Functions

**Figure 3:**
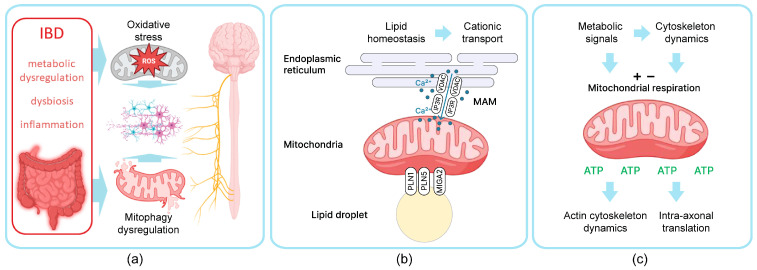
Mitochondrial functions’ involvement in GBA imbalance in IBD. (**a**) Mitochondrial health is highly crucial for neural cells’ function being subject to IBD-related pathological processes. (**b**) Lipid droplets and mitochondrion-associated membranes’ role in mitochondrial functions and calcium transport regulation. MAM—mitochondria-associated endoplasmic reticulum membrane, IP3R—inositol triphosphate receptor (a membrane glycoprotein complex that acts as a Ca^2^⁺ channel), VDAC—voltage-dependent anion channels, PLN1 and PLN5—surface proteins of lipid droplets involved in the contact of lipid droplets with mitochondria, MIGA2 is an outer mitochondrial membrane protein that directly links mitochondria to lipid droplets. (**c**) Mitochondria regulate actin filament dynamics that are crucial for intracellular junctions’ integrity through ATP production. Mitochondrial respiration also promotes mitochondria-associated intra-axonal translation of actin regulatory proteins involved in axonal branching. Both the mitochondria-dependent regulation of actin dynamics and intra-axonal translation are regulated by the modulation of mitochondrial respiration by metabolic signals and cytoskeleton dynamics. Blue arrows indicate the directions of influence between the interconnected processes shown in the schemes.

Currently, an increasing number of studies identify mitochondria as a crucial component of the GBA [199,200]. In IBD pathogenesis, mitochondrial dysfunction significantly contributes to the disruption of the intestinal epithelial barrier [90,201]. Mitochondria play a central role in cellular energy supply, ATP production, neural signal transduction, actin cytoskeleton dynamics (Figure 3); they are also involved in the homeostasis of cellular cations such as calcium and potassium, thereby participating in the regulation of various signaling pathways that either prevent or initiate inflammation and cell death [202,203,204]. Mitochondria are believed to adapt to intracellular changes in energy balance to maintain homeostasis. However, when metabolic dysregulations exceed the adaptive capabilities, mitochondrial dysfunction occurs, resulting in decreased energy production, the generation of reactive oxygen species (ROS), and oxidative stress-induced apoptosis [105,205]. Given the pivotal roles that mitochondria play in the physiology of neural cells, understanding the mechanisms of mitochondria involvement in GBA dysregulation in connection with IBD-related pathological processes, including metabolic dysregulation, could open new avenues for IBD research and therapy (Figure 3a).

### 3.1. Lipid Homeostasis Interrelation with Mitochondrial Functions

Lipid metabolism begins with the emulsification and absorption of lipids in the intestine and continues through their uptake and transport into cellular organelles, including mitochondria [130,206]. Depending on the availability of metabolic substrates, energy balance, and endocrine signaling, fats are either stored in lipid droplets within adipose tissue or oxidized in mitochondria and peroxisomes. Mitochondria are one of the main compartments for the synthesis of PL, such as phosphatidylethanolamine, which is the substrate for biosynthesis of PC, the main phospholipid of membranes and lipid droplets [104]. Unlike the transfer of free fatty acids (FFAs) from lipid droplets to mitochondria for energy production, mitochondria–lipid droplet contact can also facilitate the transfer of lipids from mitochondria to lipid droplets. Both mitochondria and lipid droplets are highly dynamic organelles. Contact sites between mitochondria and lipid droplets have been shown to support the expansion of lipid droplets by increasing ATP synthase-dependent triacylglycerol (TAG) synthesis or by stimulating lipid droplet transport [207,208]. It was hypothesized that atypical contacts between mitochondria and lipid droplets increased under conditions of high energy demand and were essential for both lipolysis and lipogenesis in response to various metabolic triggers [209]. Several members of the nuclear receptor superfamily influence all aspects of lipid metabolism [210]. PPARs and liver X receptors (LXRs), acting in concert with PPARγ coactivator 1α (PGC-1α), regulate insulin sensitivity and lipid processing [211,212,213]. These receptors are the focus of intensive pharmacological research aimed at expanding the arsenal of small molecule ligands for the treatment of diabetes and metabolic syndrome [210].

Increasing evidence suggests that mitochondria, endoplasmic reticulum (ER), and lipid droplets are functionally interconnected to facilitate the exchange of small molecules and metabolites through membrane contact sites. These contact sites are critical for maintaining organelle function and overall cellular homeostasis, including lipid homeostasis [214,215]. Current research highlights the importance of contact sites between mitochondria and the ER, as well as between mitochondria and lipid droplets, in regulating cellular lipid metabolism [216]. For instance, under starvation conditions, cells adapt by switching their metabolism from glycolysis to fatty acid oxidation. This metabolic shift is primarily achieved through the breakdown of lipid droplets and the subsequent transport of FFAs into the mitochondria for β-oxidation [217]. Given the chronic digestion and absorption impairments in IBD [218], ENS cells may enter the starvation condition. Additionally, cells can activate autophagy as an adaptive response to starvation, breaking down organelles and cell membrane proteins to generate biomolecules, such as amino acids and FFAs, necessary for survival [219]. However, excessive FFAs can be toxic to cells, generating ROS and mitochondrial damage leading to cell death due to lipotoxicity [220,221]. In the nervous system, autophagy is the most important mechanism for regulating cellular functions and neuronal homeostasis; impairments in autophagy are associated with many neurodegenerative diseases [222,223,224]. Both constitutive and stress-induced autophagy in neural cells is involved in the control and renewal of damaged mitochondria ER and other organelles [219]. Lipid homeostasis also has a significant impact on autophagy processes in neural cells [225], and the related mitochondrial dysfunction in IBD may be one of the clues to the GBA dysregulation in IBD.

### 3.2. The Role of Lipid Droplets and Mitochondrion-Associated Membranes in Mitochondrial Functions and Pathology

Mitochondria contain most of the enzymes necessary for triglyceride metabolism, emphasizing their close functional connection with lipid substrates. Lipid droplets are dynamic intracellular organelles that vary in size and primarily store TAG and sterol esters as sources of bioenergy [208]. Lipid droplets are typically located near the rough ER, and lately, their regulatory role is emerging, particularly in lipid metabolism and energy regulation [226]. Lipid droplet accumulation in nervous cells has been widely shown as the characteristic trait upon neuroinflammation and neurodegenerative processes [227]. Despite the lack of research on lipid droplets in IBD-related processes, lipid droplets in the enterocytes are considered as a functional link between lipid homeostasis and inflammatory processes [228]. Two types of interactions between lipid droplets and mitochondria are distinguished: dynamic contact, where lipid droplets bind to mitochondria via protein complexes, and stable contact, where proteins facilitate a strong and stable membrane attachment of mitochondria to lipid droplets [229]. Proteins involved in the contact between mitochondria and lipid droplets include the surface proteins of lipid droplets PLN1, PLN5, and the outer mitochondrial membrane protein MIGA2, which directly connects mitochondria with lipid droplets (Figure 3b). These contacts promote the synthesis of fatty acids, their conversion to triglycerides, and their accumulation in lipid droplets [209]. Peridroplet mitochondria, which are located in close proximity to lipid droplets, have a distinct biochemical profile compared to the rest of the cytosolic mitochondria population [209]. The fusion of lipid droplets with the mitochondrial matrix is thought not only to facilitate the use of lipids for metabolic needs but also their accumulation, as the mitochondrial matrix contains most of the enzymes of the tricarboxylic acid cycle, which remain active in peridroplet mitochondria [209,230]. The fusion of mitochondria with lipid droplets, observed in macula cells of the human vocal cords, suggests the use of triglycerides from lipid droplets for the cell’s metabolic needs [231]. Impaired glycolysis, characteristic of intestinal tissues in IBD [232], can be assumed to secondarily inhibit mitochondrial metabolism due to a decrease in the amount of pyruvate entering the glycolytic pathway. This inhibition, in turn, may activate transcription factors and corresponding enzymes that promote more active oxidation of fatty substrates, as well as stimulate mitochondrial biogenesis necessary to restore ATP production and compensate for energy deficits [233]. Increased lipid droplet biogenesis was found in association with oxidative stress and elevated ROS production [234]. The recent elegant study in a mouse model demonstrating the involvement of the GBA showed an association between targeting ROS in the intestine and behavioral changes [235].

The outer membrane of mitochondria and the membrane of the adjacent ER can form common areas, known as mitochondrion-associated membranes (MAMs), which are essential for proper communication between these organelles (Figure 3b). Currently, MAM contacts are associated with the regulation of metabolism, autophagy, aging, and the production of ROS [236]. One of the primary functions of MAMs is the transport of calcium from the ER to mitochondria, allowing fine regulation of organelle activity and their role in executing physiological and pathological signals within the cell [237]. The protein composition of MAMs is dynamic and depends on the conditions and metabolic activity of the cell; according to proteomic data, MAMs can include more than 1000 proteins [238]. The structure and function of MAMs are critical for many cellular processes. Recent studies have shown that ion transport regulation is supported by the transmission of signals between the ER and mitochondria through MAMs by several proteins located within these structures. These proteins include those encoded by the PARK genes and some proteins associated with neurodegeneration, such as huntingtin and presenilin [239]. The alteration of MAMs contributes to pathogenesis features such as autophagy dysregulation, mitochondrial dysfunction, oxidative stress, and more recently, neuronal death. These alterations are associated with neurodegenerative diseases such as Parkinson’s disease, Alzheimer’s disease, and Huntington’s disease [103,240,241].

### 3.3. The Mitophagy Regulation Importance in Neuronal Cell Health and Function

In neuronal cells, the efficient removal of damaged mitochondria through mitophagy, a highly conserved cellular process, is fundamental for maintaining mitochondrial and metabolic homeostasis, energy provision, and neuronal survival [242,243]. In healthy tissue, damaged and dysfunctional mitochondria are removed by mitophagy, preventing the accumulation of defective organelles and the development of pathological effects [242,244]. Maintaining mitochondrial function is crucial for cellular homeostasis and viability, and cells have developed a broad arsenal of quality control mechanisms to detect and eliminate defects in mitochondrial activity [244]. These mechanisms include mitochondrial biogenesis control, integrated stress response, fission and fusion events, and the selective autophagic removal of damaged or excess organelles via mitophagy. Defects in mitophagy, particularly in neuronal cells, have been found in association with neuropathology, including Alzheimer’s, Parkinson’s, and Huntington’s diseases [245,246]. Mitophagy is mediated by the major proteins PTEN-induced kinase 1 (PINK1) and Parkin [247]. In normal mitochondria, PINK1 resides on the outer mitochondrial membrane but is degraded by the inner mitochondrial membrane protease PARL, suppressing the mitophagy signal [247]. However, in damaged mitochondria with reduced membrane potential, PINK1’s amount was shown to be elevated and recognized by the Parkin protein, an E3 ubiquitin ligase, marking the mitochondria for subsequent degradation [248]. Most Parkin ubiquitination targets are localized within the mitochondria. During the early stage of mitophagy, Parkin translocates to dysfunctional mitochondria, activates PINK1, and ubiquitinates outer mitochondrial membrane (OMM) proteins [249]. During mitophagy induction, PINK1 accumulates on the OMM of damaged mitochondria, where it recruits and activates Parkin through phosphorylation. Subsequently, the factor p62 binds to ubiquitinated mitochondrial proteins, resulting in the encapsulation of mitochondria by LC3 autophagosomes, which are then degraded with the lysosomes involvement [250]. Animal models have shown that knockout of key genes responsible for mitophagy, particularly Atg5 or Pink1, leads to an increase in ROS levels [251]. The accumulation of a large number of dysfunctional mitochondria contributes to cell damage, a further increase in ROS levels, and a general inhibition of cellular energy production [219,243]. Impaired mitophagy results in the accumulation of damaged mitochondria and cellular dysfunction, contributing to neurodegeneration [244,246,252].

Mitophagy ensures the removal of dysfunctional mitochondria through several pathways that control, initiate, and facilitate this process. Mitophagy disruptions can adversely affect neuronal functions, as functional mitochondria are crucial for neurotransmitter synthesis, release, and reuptake at the synapse [243,244]. The accumulation of damaged mitochondria can lead to synaptic dysfunction and neurodegeneration, whereas mitophagy maintains synaptic integrity by eliminating damaged organelles. Damaged mitochondria can also be removed via autolysosomes, and if mitophagy is blocked, mitochondria can induce cell death (apoptosis and necrosis) [243,253]. Signal transduction between microglia, astrocytes, and neurons, which is essential for glial cell function, is also modulated by mitophagy-related pathways. Transcellular mitophagy, a process in which cells release mitochondria to be engulfed and degraded by neighboring cells, has been demonstrated in the brain [254]. For instance, glial cells can transfer mitochondria to neurons during a stroke. Dysregulation of this process can lead to neuroinflammation and loss of proteostasis [255]. Receptor-mediated transcellular mitophagy, activated under specific conditions, is driven by mitochondrial receptor proteins located on the outer or inner mitochondrial membrane containing various motifs. Although the process of transcellular mitophagy is not yet well understood and requires further study, it holds significant implications for neuronal health. Mitophagy and mitochondrial health control are valuable questions requiring further research in the context of the GBA [202,256]. Maintaining a functionally active mitochondrial network and the approaches to regulate the population of these organelles in IBD-related conditions could be ultimately essential for the GBA function.

Thus, understanding the roles of mitochondrial functions in the GBA could reveal novel insights into the IBD pathophysiology and related dysfunctions, leading to the development of new therapeutic strategies aimed at preserving or restoring mitochondrial health in IBD. The interactions between lipid droplets and mitochondria, particularly through membrane contact sites, are believed to be crucial for the regulation of lipid metabolism and the maintenance of cellular homeostasis. Further research into the roles and regulation of these processes within the context of GBA may provide valuable insights into restoring metabolic pathways in IBD.

## 4. Cationic Transport Dysregulation

**Figure 4:**
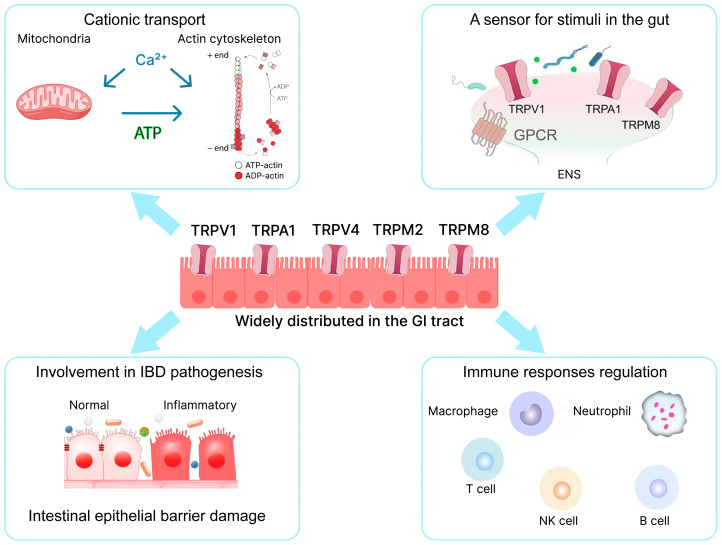
TRP ion channels’ functions in the gut enterocytes and neurons. Blue arrows indicate the key effects of TRP-mediated cationic transport dysregulation: calcium transport and calcium deficit significantly affects mitochondrial function’s and actin dynamics in both enterocytes and neurons; TRP ion channels perform crucial functions in enteric nervous system (ENS) neurons and regulate calcium transport and response to various signals; calcium transport affects gut epithelial integrity, and calcium deficit induces “leaky gut” syndrome; TRP ion channels and cationic transport regulate immune responses via immune cells receptors’ signaling.

Dysregulation of cationic balance is a crucial factor in the pathogenesis of numerous diseases, including those affecting the nervous system. The study of the roles of cations (Zn^2^⁺, Ca^2^⁺, Na⁺, K⁺, Mg^2^⁺) in CNS function remains highly relevant both under normal conditions and in pathological processes [257,258,259,260]. Calcium ions (Ca^2^⁺) play a decisive role in maintaining physiological and biochemical processes within cells. As a vital intracellular messenger, Ca^2^⁺ is actively involved in numerous physiological functions, including neuronal excitation, muscle contraction, blood clotting, and enzyme activation. It also plays roles in cell differentiation, apoptosis, the immune response, and tumorigenesis. This broad range of functions necessitates a homeostatic regulatory system [261]. It is also involved in cell differentiation and apoptosis, in the immune response, and plays a significant role in tumorigenesis [262,263]. This broad range of functions necessitates a homeostatic regulatory system [264]. The CNS is particularly dependent on calcium homeostasis, and its deregulation is closely associated with several diseases, notably neurodegenerative and inflammatory diseases [260], including IBD [265]. It is highly plausible that Ca^2^⁺ homeostasis plays a significant role in the GBA (Figure 4).

Modulating calcium influx into cells and targeting calcium-mediated signaling pathways may represent promising therapeutic approaches for these diseases. In this context, calcium channels are considered potential targets in IBD [266,267,268]. Intracellularly, three major groups of channels are responsible for releasing calcium from the ER: ryanodine receptors (RyR) [269], inositol-3-phosphate receptors (IP3R) [264,269], and two-pore channels (TPC) [270]. Several calcium channels within the plasma membrane are also crucial for CNS cell function, including voltage-gated calcium channels (VGCC) [271], ionotropic glutamate receptors [272,273], calcium release-activated calcium (CRAC) channels [274], purinergic P2X receptors [275], and transient receptor potential (TRP) ion channels [276]. Understanding and targeting Ca^2+^ channels and their regulatory pathways could provide new insights and therapeutic opportunities for managing GBA function in IBD, underscoring the importance of maintaining cationic balance in both gut and mental health.

### 4.1. TRP Ion Channels’ Role in the Inflammation and ENS Dysregulation in IBD

TRP ion channels are a large group of cation channels through which cells respond to various external stimuli. These channels are sensitive to temperature, tactile, and pain stimuli, are involved in mechanical and taste sensitivity, visual perception, and play an important role in the immune response and nociception. TRP ion channels are widely expressed in the central and peripheral nervous systems, as well as in non-neuronal cells such as those of the skin, bladder, pancreas, and spleen [277,278,279,280,281]. The currently known TRP ion channels form a large and heterogeneous superfamily, numbering more than 28 types and subdivided into subfamilies. In mammals, there are six subfamilies: TRPC (canonical), TRPV (vanilloid), TRPM (melastatin), TRPA (ankyrin), TRPP (polycystin), and TRPML (mucolipin) [280,282,283]. Accumulating evidence from clinical and animal studies supports the significant contribution of TRP ion channels to the pathophysiology of IBD. In this regard, they represent an attractive target for developing new therapeutic approaches [266,268,284,285,286]. TRPV1, TRPV2, TRPV4, TRPA1, TRPM2, and TRPM8 have been shown to be involved in the mechanisms of chronic inflammation and pain observed in IBD [287,288,289,290,291]. In the gastrointestinal tract, TRP ion channels are expressed in primary afferent sensory neurons and enteric neurons that jointly innervate the gastrointestinal tract [266,292,293]. TRP channels are also present in gastrointestinal non-neuronal cells, including enterocytes and enteroendocrine cells, such as enterochromaffin cells [294,295].

The activation of TRPV1, TRPA1, TRPV4, and TRPM8 have been shown to mediate the release of immunomodulatory neuropeptides by afferent neurons, including calcitonin-gene-related peptide (CGRP) and substance P (SP), which induce inflammatory responses [267,296,297]. The alterations of expression levels of these ion channels have been detected in IBD patients, as well as in mouse colitis models [267,268,289,298,299]. TRPV1 and TRPA1 ion channels are the most studied in the context of their involvement in intestinal inflammation. Both in IBD patients and rodent models, a significant change in the levels (both mRNA and protein) of TRPV1 and TRPA1 has been shown, which may indicate an important role of these ion channels in the inflammatory response regulation [300]. TRPV1 is hypothesized to be responsible for neuropathic inflammatory pain [300,301], as its activation promotes an increase in intracellular Ca^2^⁺ concentration and subsequent release of neuropeptides SP and CGRP from afferent sensory neurons, along with key proinflammatory cytokines [302]. TRPA1 is known to be involved in inflammatory reactions and the development of mechanical and chemical hypersensitivity in the colon [303]. The activation of TRPA1 may have a protective effect in IBD by reducing the expression of some proinflammatory neuropeptides, cytokines, and chemokines [300]. TRPV4 mRNA expression is increased in IBD patients [304], and selective blockade of TRPV4 in a mouse model of ulcerative colitis reduced intestinal inflammation and relieved pain [305]. A review summarizing data of experimental and clinical studies concluded that the TRPV4 ion channel could be considered as a therapeutic target for IBD [306].

The members of the TRPM subfamily have also been shown to be involved in IBD. Heat-sensing TRPM2 is involved in inflammatory pain and promotes visceral hypersensitivity by stimulating a range of immune functions [307]. In the gastrointestinal tract, TRPM2 is expressed mainly in mucosal macrophages and mast cells [308]. In a rat model of ulcerative colitis, the increased expression of TRPM2 was detected in the colon, and TRPM2 inactivation reduced inflammation in a mouse model of DSS-induced colitis [288]. TRPM8 ion channel activation by the agonist icilin in a mouse colitis model demonstrated strong anti-inflammatory and analgesic effect [290]. These effects may also occur due to icilin suppressing activity on another ion channel, TRPV1, which is a nociceptor that can be co-expressed with TRPM8 [309].

### 4.2. TRP Ion Channels and Gut Microbiota

Due to their immunomodulatory function through neuropeptides and immune cells, TRP channels are associated with immunity and gastrointestinal inflammation. IBD is closely associated with changes in intestinal microbial composition and metabolism, suggesting that the interaction of the gut microbiota with the CNS may occur through receptors such as TRP ion channels [294,310,311]. ENS sensory neurons innervating the intestine abundantly express TRP channels [266]. An increasing number of studies aim to identify the relationship between TRP ion channels and the intestinal microbiota [311,312]. For instance, Perez-Burgos et al. found that *Lactobacillus reuteri* dose-dependently reduced capsaicin-induced activation of jejunal afferent nerves in mice [313], suggesting that TRP channels may interact with gut microbiota and related metabolites, thereby influencing intestinal homeostasis. Interestingly, TRPV1 denervation by high doses of resiniferatoxin caused a decrease in bacterial metabolites and affected bacterial abundance in the gut [312]. In a study examining the possible interaction of TRPA1 and TRPV1 ion channels (involved in pain perception) with the gut microbiota, Nagpal et al. showed that *Trpa1^−/−^* and *Trpv1^−/−^* knockout mice were as different in microbiota composition from each other and from wild-type mice [311]. Further research into the mechanisms of the relationship between TRP ion channels and the intestinal microbiota and microbial metabolites may provide new insight into the role of these channels in physiological processes in the intestine, the GBA, and the pathophysiology of pain that occurs in IBD.

TRP cation channels play a significant role in the pathogenesis of IBD, through cation transport regulation and interactions with the intestinal microbiota. Given this, as well as the established involvement of these ion channels in neurodegenerative and neuroinflammatory diseases, it is plausible that TRP ion channels constitute a crucial link in the regulation of the GBA. Understanding the specific mechanisms by which TRP ion channels influence both gut and brain function could provide valuable insights into the interconnected nature of gastrointestinal and neurological health. This underscores the potential of targeting TRP ion channels in developing therapeutic strategies for IBD and related GBA dysregulation.

## 5. Cytoskeleton Dysregulation

**Figure 5:**
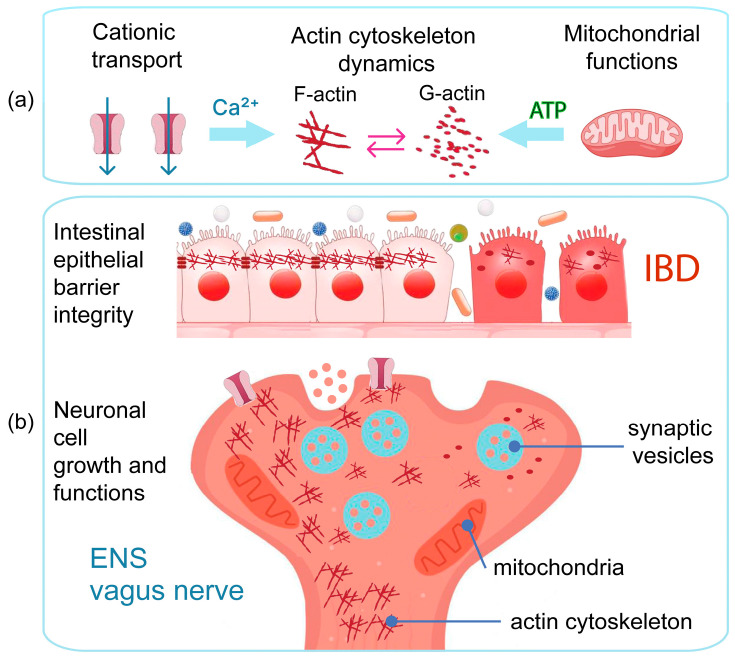
Actin cytoskeleton dynamics involved both in the intestinal barrier integrity maintenance and neuronal cell functions. (**a**) Actin cytoskeleton dynamics and actin filament formation depends on mitochondrial ATP production and sufficient Ca^2+^ cellular levels. Blue arrows show influence of ATP and Ca^2+^ levels on the assembly and disassembly of actin filaments (F-actin) from monomeric globular actin (G-actin). (**b**) Actin cytoskeleton involved in intestinal barrier integrity maintenance via intercellular junctions’ stabilization. In neuronal cells, actin cytoskeleton is essential for proper vesicle formation, transmembrane receptors’ positioning and action, axonal growth, mitochondrial movement within axons, and mitophagy.

The actin cytoskeleton plays an essential role in enterocytes maintaining the integrity of the intestinal barrier and performs vital functions in neurons [314,315,316,317,318,319]. Actin is one of the highly conserved and abundant elements of the cytoskeleton that can form highly ordered and highly dynamic linear bundles, two-dimensional networks, and three-dimensional gels depending on cellular functions and signals [320,321]. Cytoskeletal F-actin is dynamically assembled from monomeric G-actin in an ATP-dependent manner, mediated by actin nucleators and crosslinkers such as formins, fascin, and ARP2/3 [322,323]. The dynamics and stability of the actin cytoskeleton are closely related to mitochondrial health and cellular cationic transport process (Figure 5a). The dynamics of actin fibers is ATP-dependent, so mitochondrial processes have a substantial impact on cytoskeletal functions (Figure 3c). Cellular responses to environmental factors, in turn, largely occur through mitochondria-cytoskeleton interactions, involving mitochondrial dynamics, positioning, and function regulated by the cytoskeletal network [324,325]. F-actin also participates in the regulation of mitochondrial ROS production, dynamics, and positioning, with myosins implicated in mitochondrial fission events [324,326]. Cytoskeletal microtubules’ growth suppresses the fission of bound mitochondria and provides transport and anchorage tracks, while PI-(4,5)-diphosphate INF2-mediated nucleation of actin filaments promotes mitochondrial fission and dynamics [327,328]. The actin cytoskeleton mediates a variety of cellular functions, especially important for both enterocytes and neuronal cells: the maintenance of cell shape and intercellular contacts, mitochondrial health and dynamics, and vesicle formation.

### 5.1. Actin Cytoskeleton Role in Neuronal Cells and GBA Function

For proper neuronal function, the actin cytoskeleton is essential for axonal growth, mitochondrial movement within axons, and the process of mitophagy [253,329]. It is also crucial for the formation of synaptic vesicles and the reuptake of neurotransmitters [330,331]. As a result of inflammation, dysregulation of cation transport, and ATP production by mitochondria in IBD, the actin cytoskeleton may be disrupted not only in enterocytes but also in the cells of the ENS, also potentially affecting the functions of the vagus nerve (Figure 5b). Although these aspects have not been studied, they may offer promising directions for understanding ENS and vagus nerve dysfunctions within the GBA in the context of IBD. Deregulated cytoskeleton dynamics, in turn, may exacerbate mitochondrial movement and mitophagy impairments in neural cells [332]. These processes are critically important for neuronal health and proper nerve impulse transmission. Exploring therapeutic approaches to restore actin cytoskeleton function in IBD may yield systemic positive effects. Besides restoring the integrity of the intestinal barrier by reestablishing intercellular contacts, it could also improve the functions of the ENS and vagus nerve within the GBA. Given the current lack of data in this area, but recognizing the existing premises, it makes sense to focus research on the role of neuronal actin cytoskeleton dysregulation in IBD. Understanding these mechanisms may provide new insights and potential therapeutic targets to alleviate GBA-related dysfunctions in IBD.

Another possible pathway of actin cytoskeleton involvement in GBA is circulating microbial metabolites, such as short-chain fatty acids, which include butyrate and propionate [333]. Gut microbiota is known to regulate gut epithelial barrier integrity in an actin-dependent manner with butyrate [334]. These compounds have also been shown to influence the integrity of the blood–brain barrier [335,336]. The brain endothelium, a major interface between circulating metabolic signals and the brain, is a critical component of the blood–brain barrier and also depends on the actin cytoskeleton state. In an in vitro model of the tight junctions, butyrate and propionate were shown to affect the actin cytoskeleton and the structure of tight junction proteins [337]. Both these short-chain fatty acids were shown to induce distinct changes in the orientation of F-actin bundles. Additionally, they increased the number of tight junction proteins and protected against LPS-induced degradation of tight junctions, thereby improving intercellular contacts integrity and also modulating mitochondrial network dynamics [337]. These findings provide a foundation for further research into the role of actin cytoskeleton function implications for the GBA in IBD.

### 5.2. Actin Cytoskeleton Dysregulation Involvement in Gut Epithelium Barrier Dysfunction in IBD

The idea of the actin cytoskeleton’s important role in the gut epithelium barrier integrity is widely supported by findings that the destabilization of the actin network with cytoskeleton-disrupting toxins, inflammatory signaling molecules, or bacterial pathogens leads to the loss of AJC, along with an actin filament breakdown [317]. The intestinal barrier function is provided by intercellular junctions (adherent and tight junctions) of enterocytes accompanied with the mucous layer [338,339,340,341]. As a consequence of intestinal inflammation in IBD, an intestinal barrier disruption occurs [342,343,344]. Or conversely, intestinal barrier dysfunction may trigger immune activation [345,346,347]. Studies in IBD patients have revealed changes in the expression of genes of AJC proteins [348,349]. Mass spectrometry proteomic studies on human samples and animal models have also identified measurable changes in actin expression in the inflamed gut [350,351]. Several actin-binding proteins, such as cofilin, Arp3, and cortactin, colocalize with reorganized contractile rings in models of “leaky gut” induced by calcium depletion [352]. The motor protein myosin II, localized in these reorganized ring structures, is essential for AJC disassembly, as evidenced by the inhibition of its function with blebbistatin blocking that process, suggesting the involvement of actin-binding proteins in AJC regulation [353]. Recent studies have shown that the depletion of the actin-related protein (ARP2/3) inhibitor, ARPIN, alters the architecture of tight and adherent junctions while increasing actin content and epithelial barrier permeability [354]. This is supported by in vivo data in a mice model showing that ARPIN level decreases in response to DSS, and the suppression of ARPIN levels is also observed in highly inflamed areas of IBD patient samples [354]. Synaptopodin, found in actin stress fibers and tight junctions in epithelial cells, has been shown to exacerbate colitis in synaptopodin-deficient mice in response to dextran sodium sulfate [334]. There is substantial evidence of the association between calcium transport via TRP ion channels and the actin cytoskeleton, demonstrating the functional involvement of calcium transport [355,356]. Cationic transport through TRP ion channels has been shown to modulate cytoskeletal rearrangements [355,356].

In neural inflammation, calcium transport is also known to be impaired [357]. TNF-α is known to induce an F-actin rearrangement and additionally promotes the phosphorylation of the myosin light chain, leading to the disruption of tight junctions [358,359,360,361]. However, in chronic IBD, pro-inflammatory cytokine levels can be unelevated, and their effects can be balanced by anti-inflammatory cytokines, raising questions about the mechanisms of intestinal permeability changes during chronic inflammation. Studies on Crohn’s disease patients and their immediate family members have revealed genetically determined increased intestinal permeability in relatively healthy individuals, which cannot be solely explained by cytokine-induced permeability changes [362,363]. Increased intestinal permeability is also characteristic of various animal models of IBD. Animals with a null mutation in the *Nod2* gene exhibit increased intestinal permeability, mucin-2 secretion deficiency, and a predisposition to intestinal inflammation [364]. The Il10-deficient mouse strain, a popular model of IBD, also shows increased intestinal permeability, with mutations in the Il10 gene associated with a predisposition to IBD in humans [365]. While the immune pathways inducing intestinal barrier loss have been extensively studied, the contribution of cytoskeletal dynamics and metabolism remains underexplored. Interestingly, mice with a predisposition to IBD due to mutations in the Muc2 gene exhibit increased intestinal permeability, behavioral changes, metabolic dysregulation, and impaired mitochondrial function [366]. This suggests that immune activation mechanisms might not be the primary cause of intestinal barrier impairments in IBD.

The disruption of the intestinal barrier inevitably leads to an immune response due to the penetration of pathogenic factors, complicating the study of the actin cytoskeleton’s role in IBD pathogenesis. However, restoring the balance of the actin cytoskeleton to recover the epithelial barrier function presents a promising direction for IBD therapy. Understanding and targeting the actin cytoskeleton dysregulation in IBD could provide novel insights and therapeutic opportunities for managing IBD and also restoring GBA functions. Regarding cytoskeletal modulation, new strategies may emerge to alleviate the chronic inflammation and neural dysfunctions associated with IBD.

## 6. Discussion: Clinical Applications and Translational Perspective

Considering recent data in patients with various forms and at different stages of IBD experiencing significant metabolic dysregulation [80,167,170,173,367,368], the extensive studies of pathophysiological mechanisms and regulatory pathway involved in the interrelations of these processes with GBA function become a highly relevant area. In this review, we systematically summarized the research data which shed light on the range of systemic effects of metabolic dysregulation in IBD on molecular and cellular processes critical for GBA functioning. The most significant effects of IBD in relation to metabolic dysregulation are the substantial lipidome deregulation [86,87] along with oxidative processes’ dysregulation and mitochondrial malfunction [82,83,84,85]. Dysregulation of the lipidome entails multiple dramatic consequences, which consist not only in systemic processes’ distortions, but also in basic cellular functions’ lesions. Lipid metabolism is extremely important in biosignaling, since major classes of lipids, such as PUFAs and phospholipids, are precursors to a spectrum of messengers that mediate metabolic regulation and also the development and control of inflammation, as well as the regulation of nervous activity and signaling processes [368]. Along with this, the disproportions of phospholipids levels and phospholipid processing impairments lead to various crucial structural and signaling consequences [369]. Thus, changes in the composition and proportions of phospholipids play a significant role in the function of membrane cell structures and the integrity of the epithelial barrier (both intestinal and blood–brain barriers). Moreover, phospholipids perform important functions in intracellular transport and other intracellular membranous structures, in particular in the organelles’ health, and to a significant extent, in mitochondrial health [369,370]. Data are just emerging identifying specific lipid indicators as clinical predictors for IBD [79,87,146]. However, this area has not yet been studied enough to offer specific targeted therapeutic approaches for metabolic dysregulation correction in IBD patients, in particular, lipidome dysregulation. However, it becomes clear that this direction contains great translational prospects, especially in relation to the GBA balance restoration in IBD.

The GBA, especially in the clinical context of IBD, is currently often considered to include the liver function, which is inevitably affected by pathological processes associated with both chronic inflammation and metabolic dysregulation [371]. In particular, BAs are coming to the fore in the therapeutic perspective. Patients with different types and stages of IBD have different bile acid profiles [171,172,173,174]. As more clinical data accumulate, the composition of BAs in serum and feces may have a diagnostic or prognostic value [372]. The microbiota have long been and are currently undoubtedly considered the most important component of the GBA, and a great amount of research shows the clinical and prognostic significance and a great therapeutic potential of the microbiota in IBD. The imbalance in the gut microbiome found in patients with neurodegenerative diseases saw a rise in the exploration of the clinical application of microbiome-based therapies involving the GBA, including prebiotics, probiotics, and fecal microbiota transplant (FMT). This direction was supported by studies showing the human gut microbiome as a major determinant of the plasma metabolome, potentially playing a more dominant clinical role than the patients’ genome [373,374,375]. Although significant overall positive effects have been shown from taking probiotics, as well as synbiotics and signaling substances produced by the microbiota, in particular on the mood and cognition in IBD and IBS patients, there is still a substantial lack of fundamental understanding of the underlying molecular pathways and cellular mechanisms to develop reliable therapeutic strategies [199,376,377,378]. As BAs perform a bactericidal function and are important components of the intestine’s innate immunity participating in the control of its bacterial composition, they are promising in relation to the regulation of the GBA. It has been shown that primary taurocholic acid is required for the development of *Clostridioides difficile* [379], whereas secondary LCA and UDCA inhibit the growth of *C. difficile* [380]. Intestinal inflammation caused by this pathogen is often observed in patients with IBD [381]. Thus, in FMT in children with IBD as a treatment of recurrent *C. difficile* infection, only a temporary restoration of the diversity of bacterial microflora was observed and by 6 months it corresponded to the donor level, whereas in children with and without IBD, during the same time, there was a decrease in primary and an increase in secondary BAs, but only in children with IBD did the level reach the donor level [381]. Similar changes in secondary bile acid levels, as well as increases in short-chain fatty acid butyrate, acetate, and propionate levels, were observed after FMT in adult patients [382]. It is likely that the ratio changes of BAs in feces are one of the mechanisms of the therapeutic effect of FMT. Secondary bile acids are currently used in complex therapy of the nervous system pathologies, for example, TUDCA has a neuroprotective, anti-apoptotic effect, and also acts as a mitochondrial stabilizer in various brain disorders, including when taken orally [383]. In addition, secondary bile acids, for example, UDCA, have anti-inflammatory, cytoprotective, and anti-apoptotic effects and are applied in therapies for a wide range of neurodegenerative diseases, including amyotrophic lateral sclerosis, Alzheimer’s disease, Parkinson’s disease, and Huntington’s disease [383]. A number of studies in both animal models and patient data suggest that UDCA also may have a therapeutic role in IBD in regard to GBA influencing intestinal microbiota [384]. Thus, oral administration of UDCA or its taurine- or glycine-conjugated species to mice with experimental colitis reduced its severity, normalized the increased ratio of *Firmicutes* to *Bacteroidetes* associated with colitis, but did not restore bacterial diversity [385]. Currently, there is a lack of therapeutic observations of the effect of BAs on the condition of patients with IBD and nervous system disorders. At the moment, there is only one study that provides clinical results confirming the involvement of gut dysbiosis and BAs metabolic alteration in psychological disorders in Crohn’s disease [171].

The next important interconnected links in the chain of GBA balance are the cation transport, actin cytoskeleton, and mitochondrial health (Figure 5). Regarding these basic cellular processes, there are currently few data that could prove their direct interconnection in IBD [386]. However, substantial impairments of all these anchors have been shown in animal models of IBD [85,89,90,387], as well as in several clinical studies [388,389]. To date, one of the well-studied candidates linking these processes within the GBA concept seems to be TRP ion channels families. TRP ion channels are widely expressed in the gastrointestinal tract and peripheral and central nervous systems, performing a number of important functions there [268,294] (Figure 4). It is known that expression or activity changes, as well as mutations in TRP genes, are often associated with a wide range of intestinal epithelial disorders, including IBD, IBS, fibrosis, visceral hyperalgesia, and colorectal cancer [390]. Therefore, TRP channels are considered potential targets for new analgesics effective within the research and development of new approaches targeting TRP to reduce inflammation and relieve pain in IBD. It has now been shown that TRP channels play a significant role in the pathogenesis of IBD through the regulation of cation transport and interaction with the intestinal microbiota [391]. Currently, the most studied in this regard are TRPV1 and TRPA1, which are known as nociceptors [392,393]. TRPV1 has been shown to be responsible for neuropathic inflammatory pain [300,301] and hyperalgesia [394]. In this context, the interesting clinical study of biopsies from IBS showed that the number of nerve fibers expressing TRPV1 in the colon was markedly increased in relation to a healthy control group, and this increase was correlated with pain severity [395]. TRPA1 is also known to be involved in inflammatory responses and the development of mechanical and chemical hypersensitivity in the colon [303]. It has been shown that the activation of TRPA1 may have a protective effect in IBD by reducing the expression of certain pro-inflammatory neuropeptides, cytokines, and chemokines [300]. Meseguer et al. reported that bacterial lipopolysaccharides could activate TRPA1 channels, causing acute intestinal inflammation and visceral pain [396]. On the other hand, it has been shown that TRP ion channels play a critical role in the dynamics of the actin cytoskeleton [356]. Both the integrity of the intestinal barrier and mitochondrial health in the intestinal cells and neural cells depend on the actin cytoskeleton dynamics [85,317,397]. Mitochondrial functions also depend strictly on both the proper cytoskeleton functioning and calcium transport within the cell. In this review, we summarized data on the involvement and interrelations between these processes in the context of the GBA function misbalance in IBD. The search for new approaches to restore the function of each of these GBA links in IBD may offer ways to achieve long-term and self-sustaining positive therapeutic effects.

## 7. Conclusions

Numerous studies in both patients and animal models highlight the extensive involvement and importance of the GBA in IBD. Despite the significant interest in the GBA and the extensive research on its role and function in IBD, many aspects of this complex relationship remain unclear. Beyond the primary components of the GBA—vagus nerve function, ENS function, and microbiota—IBD leads to impairments in metabolic regulation, mitochondrial function, cationic transport, and cytoskeleton dynamics in intestinal cells. These mechanisms are also crucial for the proper functioning of neuronal cells. Acute and chronic inflammation, dysbiosis, and impaired digestion and absorption in IBD result in significant metabolic dysregulation. This dysregulation majorly affects the lipidome and cellular energy cycles, which are essential for the nervous system functions. Additionally, due to multiple changes in regulatory metabolic signals, cation transport and mitochondrial functions are disrupted in IBD. Consequently, actin cytoskeleton dysregulation might occur in both enterocytes and nervous system cells. Proper calcium transport is critical for neuronal and mitochondrial functions and is vital for maintaining the integrity of the epithelial barrier. Therefore, all the described processes are interconnected through direct and reverse regulatory pathways, potentially leading to the self-reinforcement of pathology. Deciphering these complex interrelations and their effects on the GBA is crucial. This knowledge may reveal new directions in the search for therapeutic targets and means to improve the quality of life for IBD patients. Despite the large number of studies, there is still a limited clinical arsenal for IBD treatment; mostly, a temporary alleviation of IBD symptoms is possible. Only a small percentage of patients stably respond to therapy, and only a small percentage achieves a complete cure, along with frequent relapses and self-reinforcement, in which GBA imbalance is shown to play a significant role. A further in-depth study of the systemic effects associated with GBA misbalance in IBD, summarized in this review, as well as the discovery of new therapeutic approaches to the restoration of impaired metabolic, cellular, and organelle functions within the GBA in IBD patients could suggest great translational prospects.

## Data Availability

No new data were created or analyzed in this study. Data sharing is not applicable to this article.
